# Characterization of Heparin Interactions With Recombinant Rodent Stabilin-2/Hyaluronic Acid Receptor for Endocytosis (HARE)

**DOI:** 10.1002/pgr2.70027

**Published:** 2025-04-07

**Authors:** Reed A. Rohr, Evan A. Schroder, Joseph D. Staab, William P. Singh, Callan J. Schroder, Grant D. Hatcher, Joshua T. McWilliams, Jiyuan Yang, Abby E. Bopp, Linda B. Fatumoju, Zhangjie Wang, Jonathan S. Dordick, Robert J. Linhardt, Fuming Zhang, Jian Liu, Edward N. Harris

**Affiliations:** 1 University of Nebraska, Lincoln, Nebraska, USA; 2 The Key Laboratory of Molecular Microbiology and Technology, Ministry of Education, College of Life Sciences, Nankai University, Tianjin, P.R. China; 3 Department of Chemical and Biological Engineering, Rensselaer Polytechnic Institute, Troy, New York, USA; 4 University of North Carolina Chapel Hill, Chapel Hill, North Carolina, USA

## Abstract

Stabilin-2 is the primary scavenger for hyaluronan (HA) and binds to over two dozen other ligands including chondroitin sulfates, heparin, oxidized/acetylated LDL, etc. Although rat liver sinusoidal endothelial cells are the preferred primary cell lines and animal for physiological studies of Stab2/HARE, the rat recombinant protein has never been characterized. Since the rat Stab2/HARE has a high degree homology to mouse Stab2/HARE which has been cloned, our hypothesis is that the rat receptor is identical to mouse and very similar to the human receptor. Rat Stab2/HARE was cloned and expressed in the FlpIn HEK293 cell line. The recombinant protein was analyzed for HA and heparin binding/endocytosis as well as synthetic heparin (Dekaparin) in a mouse knockout model. The secreted ecto-domain was also created for surface plasmon resonance analysis. The physical structure of rat Stab2/HARE is different than human in that the small isoform is not expressed as robustly and reduction of the protein results in what is likely two physical conformational forms. Rat Stab2/HARE binding strength with HA is weaker when compared to human Stab2/HARE, but rate of endocytosis is higher. Heparin-Stab2/HARE bonding strength is similar to human, though endocytic rate tends to be higher. Metabolism of Dekaprin is delayed in a Stab2KO mouse model and affects liver sequestration of this drug. Rat Stab2/HARE has similar properties as the human Stab2/HARE with the exceptions that the rat recombinant protein has a different physical structure and has an increased HA and heparin internalization rate.

## 1 | Introduction

Heparin/heparan sulfate and hyaluronan (HA) are glycosaminoglycans that are involved with many biological activities. Hyaluronan (HA) is a polymer of the disaccharide, glucuronic acid and N-acetylglucosamine, which is a crucial component of the extracellular matrix [1]. HA is ubiquitous throughout the human body, but has higher concentrations in the bursal tissue thereby protecting the joints and composing the vitreous humor [2–4]. Systemic clearance of HA was first reported by Fraser and colleagues in 1981 using a rabbit animal model in which they observed that HA accumulated mostly in the nonparenchymal liver cells [5]. Closer scrutiny of the nonparenchymal cells revealed that the liver sinusoidal endothelia was responsible for well over 90% of the HA uptake and degradation of HA [6]. The pathway of HA catabolism was more clearly established in that freed HA polymers are exposed to the lymphatic vessels where much of the breakdown takes place, followed by the blood where any remaining HA is taken up by liver [7].

HA uptake by cells and tissues was observed to be a saturable event [6]. Evidence that there was a membrane receptor associated with HA binding and uptake and that this receptor recycled was reported by McGary and coworkers [8]. HA binding was calcium independent [9] and readily degraded by liver sinusoidal endothelial cells (LSECs) [10]. In the 1990s, there were two independent groups working in parallel to clone and characterize this HA-binding receptor. The first group was led by Bard Smedsrod who worked initially with Fraser and Laurent. Some of the initial characterization of receptor activity was confirmed in the 1980s and by 1999, this group published a paper in Hepatology demonstrating that proteins of specific size bound HA and confirmed this using antibodies generated against the hyaluronan receptor from column purified proteins [11]. Their German collaborators further refined this study published in 2002 in which their 270-kDa human receptor was formally named Stabilin-2 [12] due to its high homology to Stabilin-1 which does not bind HA. The second group, led by Paul Weigel, purified a 175-kDa receptor from rat LSECs by column chromatography and developed antibodies that were active against both 175-kDa and 300-kDa receptors in rat that also cross-linked with human receptors [13, 14]. The smaller 175-kDa receptor, HA receptor for endocytosis (HARE), was cloned into the SK-Hep cell line to prove that the cloned receptor was active [15]. In 2003, Zhou et al. published a paper describing the purification and molecular identification of the 175-HARE [16] with some of these points challenged by B. Smedsrod et al. [17]. One of the issues was the characterization of the receptor in which the human form was a monomer represented by the Smedsrod group and a multimer represented by the Weigel group. Through the intervening years, Stabilin-2 expressed in mouse and human was known to be a large monomeric protein receptors that bound HA and the smaller isoform kept the moniker, HARE [18]. The smaller isoform is a proteolytic product of the larger isoform [19–21] as its expression always appears from a recombinant cDNA encoding only the large isoform.

Heparin, like HA, is a polymer of monosaccharides that are heavily sulfated, more so than heparan sulfate, with a much greater rate of heterogeneity in terms of sugar modification and polymer length [22]. Heparin is produced in mast cells with the highest concentrations in the intestinal mucosa. Heparin is a proteoglycan that is covalently attached to the host protein, Serglycin which is released during mast cell activation. Heparin was discovered in the early twentieth century and eventually came into medical use as an anticoagulant [23]. Although there are a number of receptors that bind with heparin in various biological capacities in different cell types [24, 25], the catabolism of heparin was not well-understood until the Stab2-heparin interaction was characterized [26]. These data combined with previous experiments demonstrated that heparin had a hepatotropic route of catabolism [27]. Further experiments, in animal models revealed that LSECs that have high expression of Stab2/HARE internalize heparin [28]. However, in these animal studies, the authors observed similar behaviors in primary cells and did not observe the rodent receptors specifically. Unfractionated heparin (UFH) was used in these reported experiments and is a refined product from porcine intestinal-derived material that is unmodified.

Despite the cloning and characterization of mouse and human HARE, no one cloned the large Stab2/HARE receptor from rat. The Weigel group had always observed it in LSEC lysates separated by SDS-PAGE, but the full-length receptor was never cloned or expressed as a recombinant protein; only the small 175HARE in SK-hep cells [15]. We hypothesized that the rat and mouse, and by extension, human Stabilin-2 are virtually identical with all the same physical characteristics and ligand binding activity. Since rats and mice are animal models for human disease, it is important and prudent to further characterize the rat Stabilin-2 and, by extension, the mouse Stabilin-2 as both receptors have very high homology. This manuscript describes the cloning and characterization of the rat Stabilin-2 (300HARE), its interaction with heparin and HA and differences between human (315HARE/190HARE) and rodent (300HARE/175HARE).

## 2 | Materials and Methods

### 2.1 | Animal Models

The Stabilin-2 knockout mouse was generated in the C57BL/6 J background strain and described by Hirose et al [29]. Both WT C57BL/6 J mice and Stab2KO mice were kept in a temperature controlled 12/12 light-dark facility that has been approved by the American Association for Accreditation of Laboratory Animal Care (AAALAC) at the University of Nebraska—Lincoln under protocol #2388. Mice were fed standard animal chow and water ad lib. All animals were housed under ARRIVE guidelines.

### 2.2 | Tail Vein Injections

Mice were inserted into a mouse tail illuminator/restrainer (Braintree Scientific, #MTI) fully alert. Fifty uL of 200 nM b-UFH conjugated with 2.5 μg/mL ^125^I-Streptavidin (SA) was injected via the lateral tailvein and allowed to circulate for 20 min. During the incubation time, the mouse was freed from the restrainer and allowed to roam in the housing cage. Mice were then anesthetized with 30% isoflurane dissolved in polypropylene glycol 200 that was placed in a cotton ball within a small containment area (200 mL crystallizing dish). Blood and liver were collected and weighed/measured, and radioactivity was evaluated by a gamma counter. Six animals of each genotype were used for each of the UFH and SA alone injections.

### 2.3 | Dekaparin Anabolism

The synthesis of Dekaparin (Figure S6) is described in Xu 2014 [30]. A dose of 0.6 mg/kg was injected subcutaneously in each mouse. At the indicated time points, mice were anesthetized with the use of isoflurane in a nebulizer and blood was collected with a mylar-wrapped capillary tube (Drummond Scientific #1-000-7500-C) via retro-orbital bleed. Blood was immediately ejected into a 1.5 mL Eppendorf tube containing 20 μL 0.5 M EDTA to prevent clotting. We collected approximately 0.3 mL (three full capillary tubes) from each mouse and mice were bled only once. Each time point contained five mice for both genotypes and all animals survived the procedure well.

### 2.4 | Purification of 12mer (Dekaparin) From Plasma

Dekaparin extraction from the plasma samples was performed with protein precipitation, proteinase digestion, DEAE column purification and heparin lyases depolymerization. The sample (30–100 μL) was mixed with 800 μL methanol, vortexed for 1 min, and allowed protein precipitation at room temperature for 10 min. The sample was centrifuged at 1000 × g for 10 min and the supernatant was discarded. The pellet was digested with 50 μL Pronase E (20 μg/mL), at 55°C for 24 h to degrade the proteins. After digestion, the solution was boiled at 100°C for 10 min and centrifuged at 14,000 rpm for 10 min to discard insoluble matters. 12mer was recovered from the digested solution using a micro DEAE-column (200 μL). DEAE column mobile phase A contained 20 mM Tris, pH 7.5 and 50 mM NaCl, and mobile phase B contained 20 mM Tris, pH 7.5 and 1 M NaCl. After loading the digested solution, the column was washed with 10 column volumes of buffer A, followed by 10 column volumes of buffer B to elute the 12mer. The eluted 12mer was desalted using an YM-3KDa spin device and the device washed three times with deionized water. Before desalting, 300 ng ^13^C-labeled 12mer internal standard was added into each sample. The spin device was reversed to recover the retentate from the membrane and washed three times with deionized water. The combined solution was dried before the digestion with heparin lyases. The 100 μL of digestion solution containing 7.5 μL of enzymatic buffer (100 mM sodium acetate/2 mM calcium acetate buffer (pH 7.0) containing 0.1 g/L BSA), and 1.25 μL heparin lyases I (2.49 mg/mL) and 2.5 μL heparin lyases II (13.6 mg/mL). The reaction solution was incubated at 37°C for 12 h. The 12mer digest were recovered by centrifugation, and supernatant were freeze-dried before the AMAC derivatization.

### 2.5 | Chemical Derivatization of Dekaparin Trisaccharide

The 2-Aminoacridone (AMAC) derivatization of freeze-dried samples was performed by adding 5 μL of 0.1 M AMAC solution in DMSO/glacial acetic acid (17:3, v/v) and incubating at room temperature for 15 min. Then 5 μL of 1 M aqueous sodium cyanoborohydride (freshly prepared) was added to this solution. The reaction mixture was incubated at 45°C for 2 h. After incubation, the reaction solution was centrifuged to obtain the supernatant that was subjected to the LC-MS/MS analysis.

### 2.6 | LC-MS/MS Analysis of the Trisaccharide

The analysis of AMAC-labeled trisaccharide was implemented on a Vanquish Flex UHPLC System (Thermo Fisher Scientific) coupled with TSQ Fortis triple-quadrupole mass spectrometry as the detector. The ACQUITY Glycan BEH Amide column (1.7 μm, 2.1 × 150 mm; Waters, Ireland, UK) was used to separate the trisaccharide at 60°C. Mobile phase A was 50 mM ammonium formate in water, pH 4.4. Mobile phase B is acetonitrile. The elution gradient as follows: 0–6 min 83% B, 6–13 min 60% B, 13–18 min 30% B. The flow rate was 0.25 mL/min. On-line triple-quadrupole mass spectrometry operating in the multiple reaction monitoring (MRM) mode was used as the detector. The ESI-MS analysis was operated in the negative-ion mode using the following parameters: Neg ion spray voltage at 3.0 kV, sheath gas at 55 Arb, aux gas 25 arb, ion transfer tube temp at 250°C and vaporizer temp at 400°C. Trace-Finder software was applied for data processing.

### 2.7 | Biotinylation of HA and UFH

Biotinylation of HA was performed according to the method of Thakar et al. [31]. Briefly for the oxime ligation via the reducing end, 4 mM HA was resuspended in 100 mM sodium acetate pH 4.5 and incubated with 3.4 mM Biotin-dPEG3-oxyamine HCl (Biosynth, Louisville, KY USA) for 2 days at 37°C in a shaker. The solution was then dialyzed against three changes of PBS with the last change going overnight. The molar amount of HA was determined by the carbazole assay [32] and the molar amount of biotin was determined by QuanTag Biotin Quantitation kit (Vector labs, Newark, CA, USA). Biotinylation of UFH was adapted from the method of Yu and Toole [33]. Briefly, 2 mM of UFH assessed by size exclusion chromatography in tandem with multi-angled light scattering (Waters Corp, Milford, MA, USA) to be around 13.5 kDa in 100 mM 2-N-morpholinoethanesulfonic acid (MES buffer, pH 5.5) was incubated with 6 mM Biotin-LC-Hydrazide and 1.3 mg/mL 1-ethyl-3-[3-dimethylaminopropyl] carbodiimide HCl overnight at 4°C under rotation. This solution was dialyzed against PBS and molar amounts of biotin and UFH were quantified similar to HA. Porcine intestinal biotinylated heparin (biotinylation at the reducing end) used as a control heparin for SPR to compare biotinylated UFH was synthesized using the following method: 2 mg heparin and 2 mg amine-PEG3-Biotin (Thermo Scientific, Waltham, MA, USA) were mixed in 200 μL water, following addition of 10 mg NaCNBH_3_. The reaction was carried out at 70°C for 24 h, after that another 10 mg NaCNBH_3_ was added, and the mixture was incubated at 70°C for another 24 h. When the reaction was finished, the product was desalted using a spin column (3000 molecular weight cut-off). The salt free biotinylated heparin was lyophilized.

### 2.8 | Iodination of Streptavidin (SA)

Pure streptavidin (125 μg) was placed in a glass 12 × 75 glass tube coated with 1,3,4,6-tetrachloro-3α,6α-diphenyl glycoluril (Iodogen) along with 50 μL water and 0.3 mCi label-free ^125^Iodine for 20 min at room temperature. The mixture was then allowed to flow through a PD-10 column to separate the labeled streptavidin from free ^125^Iodine and the eluate was collected in 0.5 mL fractions. Fractions were evaluated for both radioactivity and protein which were pooled together. Final protein content was determined by the Bicinchoninic assay (BCA). For all assays, 200 nM biotinylated HA or UFH was conjugated with 2.5 μg/mL ^125^I-SA using a benchtop orbital tube shaker (Scilogix, Rocky Hill, CT) at a rate of 200 rpm.

### 2.9 | Endocytosis and Binding of HA/UFH

All endocytosis and binding experiments were performed similarly to previous reports [34]. Briefly, for endocytosis, cells were plated in 24-well plates at a confluency of 70% and allowed at least 24 h to recover and grow up to 90% confluency. On the day of the experiment, 0.3 mL of endocytosis media per well along with the equivalent amount of 200 nM of b-GAG was combined with 2.5 μg/mL ^125^I-SA. For mixing the biotin and SA conjugates, the amount of b-GAG and ^125^I-SA was calculated and then they were mixed in a 0.5 mL DMEM as a concentrated mix and then diluted for the assay at an equivalent of 200 nM of b-GAG combined with 2.5 μg/mL ^125^I-SA. Endocytosis was allowed to proceed at 37°C in a tissue culture incubator at the specified times. Cells were harvested by washing three times in cold HBSS and lysing the cells in 0.3 M NaOH. The amount of radioactivity was measured by a gamma counter and the protein in cellular lysates was measured by the Bradford assay (Thermofisher, La Jolla, CA). Binding assays were very similar to endocytosis assays except that media components were chilled on ice and cells were incubated on ice for 1 h. To assess total binding, the addition of 0.055% digitonin dissolved in DMSO was added to the chilled media. Digitonin permeabilizes the plasma membrane and allows access of the ligand to endosomal-bound receptors [9].

### 2.10 | Binding Kinetics Measurement of Interaction Between HA/Heparin and Protein sr175 Using SPR

Binding kinetics measurement was performed with Biocore T200 system (Cytiva, Uppsala, Sweden). To prepare the HA or heparin chip, biotinylated HA or heparin was immobilized to a streptavidin (SA) chip based on the manufacturer’s protocol. In brief, 20 μL of biotinylated HA (0.1 mg/mL) in HBS-EP+ running buffer (0.01 M HEPES, 0.15 M NaCl, 3 mM EDTA, 0.05% surfactant P20, pH 7.4) (Cytiva, Uppsala, Sweden) was injected overflow cell 2, and biotinylated heparin was injected over flow cell 3 of the SA chip at a flow rate of 10 μL/min. The successful immobilization of HA or heparin was confirmed by increased base-line shift (648 RU for HA, 732 RU for heparin) after the injection. Saturated biotin in HBS-EP+ buffer was injected onto flow cell 1 (FC1) as control. To measure the binding kinetics, different dilutions of protein samples in HBS-EP+ buffer were injected to the chip at a flow rate of 30 μL/min. At the end of each sample injection, HBS-EP+ buffer was flowed over the sensor surface to measure dissociation. After dissociation phase, the sensor surface was fully regenerated by injecting with 30 μL of 2 M NaCl. The sensorgrams (response [RU] as a function of time [s]) were monitored at 25°C. Binding kinetics (ka, kd) and affinity (K_D_) were calculated by globally fitting the sensorgrams using a 1:1 Langmuir-binding model from T200 evaluation software (Cytiva, Uppsala, Sweden).

### 2.11 | Direct Binding With ELISA-Like Assay

The s190 and sr175 proteins were purified using metal affinity chelate chromatography methods described in Harris et al. [35]. For this assay, both proteins were 4 μg/mL in coating buffer (15 mM Na_2_CO_3_, 36 mM NaHCO_3_, pH 9.5) and plated individually in F8 polysorp wells (Nunc, Denmark) and allowed to bind overnight. The wells were then aspirated and blocked with TBST blocking buffer (Tris-buffered saline: 154 mM NaCl, 10 mM Tris-HCl pH 7.2, 0.1% BSA, 0.025% Tween-20) for at least 1 h. All solutions were made with blocking buffer. After washing the wells out three times, 200 nM biotinylated heparin (UFH) was incubated with s190 or sr175 in 0.4, 0.8, 1.2, 1.8, and 2.4 M salt solutions made in TBST or in plain TBST for 2 h. After washing three times, the wells were incubated with 2.5 μg/mL ^125^I-Streptavidin for 1 h, washed and cleaned out with 200 μL 2% SDS solution. The SDS solutions from each well were placed in individual polystyrene 12 × 75 tubes and counted with a 1470 Wizard gamma counter (Wallac/LKB instruments, Gungahlin, Australia). To determine whether the salts dislodged proteins from the wells, we detected the amount of bound protein by primary rabbit anti-V5 antibody (Bethyl labs/Fortis Bio, Montgomery TX) followed by anti-rabbit alkaline phosphatase secondary (Cell Signaling Technology, Danvers, MA) and incubating in development buffer (100 mM Glycine, 1 mM MgCl_2_, 1 mM ZnCl_2_, pH 10.4). Color development was measured by a SpectroMax M2 plate reader (Molecular Devices, San Jose, CA) at an absorbance value of 420 nm.

### 2.12 | Cloning and Cell Line Generation

A fresh rat spleen (100 mg) was homogenized in 1 mL ice-cold Trizol reagent and RNA extracted according to manufacturer’s instructions. cDNA was generated using the dT18 primers with the One Step kit. Three overlapping fragments of Rat Stab2/HARE were amplified using primers designed by NEBuilder Assembly Tool (NEB, Ipswich, MA, USA). Fragments were assembled along with pcDNA5/FRT/V5/6xHis plasmid which was verified by sequencing and subject to CsCl purification before transfection. Three million Flp-In HEK293 cells were plated in 100 mm cell culture dishes 2 days before transfection. The transfection mixture consisted of 750 μL serum free media, 9 μg pOG44, 1 μg rat Stab2/HARE, and 20 μL Lipofectamine 2000. Medium was replaced on the cells every 2 days for the first week after transfection and colonies were selected 2–3 weeks posttransfection. Individual clones were grown in 12-well plates and selected based on cellular morphology, replication rate, and protein expression. All recombinant proteins were detected by anti-V5 antibodies (Bethy labs/Fortis Life Sciences, Montgomery Tx).

### 2.13 | Mutagenesis of C2469

The full-length *Stab2/HARE* cDNA was initially cloned in pcDNA5/FRT/V5/6xHis (Life Technologies, Carlsbad, CA, USA) and mutagenesis of C2469 was performed using In-Fusion technology (Takara Bio, Kusatsu, Shiga, Japan). Primers used for swapping out the C to a V were the following: 5′-TATATTCGCTGCCGTCGTCCTGGTCACTG-3′ and 5′-ACGGCAGCGAATATACCTGTCCCCAGGCCAG-3′. All experimentation was performed in transiently transfected cells.

### 2.14 | Mass Spectrometry of Coomassie Stained Protein Bands

Three gel bands in duplicate were excised, cut into small cubes, and placed in microcentrifuge tubes. The gel samples were re-suspended in 100 mM ammonium bicarbonate. One part of each band was digested with MS-grade Pierce trypsin (cleavage sites KR), while the other part was digested with V8 enzyme (cleavage sites DE) overnight at 37°C. Before digestion, the samples were reduced with 10 mM DTT at 56°C for 30 min and alkylated with 50 mM iodoacetamide at room temperature for 25 min. Peptides were desalted using PepClean C18 spin columns (Thermo Scientific, La Jolla, CA, USA) and re-suspended in 2% acetonitrile (ACN) and 0.1% formic acid (FA). A total of 500 ng of each sample was loaded onto a trap column (Acclaim PepMap 100, 75 μm × 2 cm C18, Thermo Scientific^™^) at a flow rate of 4 μL/min, and then separated on a Thermo RSLC Ultimate 3000 (Thermo Scientific^™^) using a Thermo Easy-Spray PepMap RSLC C18 column (75 μm × 50 cm, 2 μm, Thermo Scientific^™^). The separation was performed with a step gradient of 4%–25% solvent B (0.1% FA in 80% ACN) from 10 to 60 min, followed by 25%–45% solvent B from 60 to 70 min, at a flow rate of 250 nL/min and 50°C, with a total run time of 70 min. Eluted peptides were analyzed using a Thermo Orbitrap Exploris 480 (Thermo Scientific^™^) mass spectrometer in data-dependent acquisition mode. A survey full scan (m/z 350–1200) was acquired in the Orbitrap with a resolution of 60,000. The normalized AGC target for MS1 was set to 300%, and the ion filling time was set to 25 ms. The most intense ions with charge states 2–6 were isolated in a 3-s cycle and fragmented using HCD with 30% normalized collision energy, and detected with a mass resolution of 15,000 at 200 m/z. The AGC target for MS/MS was set to 50%, with ion filling time set to auto for 30 s and a 10 ppm mass window. Protein identification was performed by searching the MS/MS data against the sequence of a single rat recombinant protein expressed in human cells, as well as the reviewed human proteome from SwissProt, downloaded from UniProt on August 7, 2024, using Proteome Discoverer 3.0 software. The search parameters included semi-tryptic peptides and V8 enzyme with a maximum of two missed cleavages and at least two unique peptides per protein. Acetylation of protein N-terminus and oxidation of methionine were included as variable modifications, and carbamidomethylation of cysteine was set as a fixed modification. The precursor mass tolerance was set to 5 ppm, and the maximum fragment mass error was 0.02 Da. The significance threshold of the ion score was determined based on a false discovery rate (FDR) of ≤ 1%.

### 2.15 | Western Blot Analysis

All protein mixtures including cell lysates or immunopreciptate2 or pure protein samples were separated by 5% SDS-PAGE (Mini-protean tetra, BioRad, Hercules, CA) and blotted (Genie Blotter, Idea Scientific, Minneapolis, MN) to nitrocellulose membranes. The molecular mass marker used in all gels was the PageRuler provided by Thermofisher (Waltham, MA, USA). Membranes were blocked with Pierce Protein-Free (PBS) blocking buffer (#37572, Thermofisher, Waltham, MA, USA) for at least 1 h before probing with anti-V5 primary antibody (Bethyl Labs, Montgomery, TX) and LiCor secondary antibodies for quantification in infrared spectrum with LiCor FC instrumentation. All original images for Western blots are provided in Figure S7.

### 2.16 | Statistical Analysis

All experimental data is expressed as mean ± stand deviation (SD) in triplicate. Some calculations were as mean ± standard error of the mean (SEM) if replicate experiments were performed at different times with different batches of ^125^I isotope. All animal number values were at least five individuals for each data point. Differences were considered significant at the level of *p* ≤ 0.05. All statistics were calculated using SigmaPlot v11.2.

## 3 | Results and Discussion

**FIGURE 1 |:**
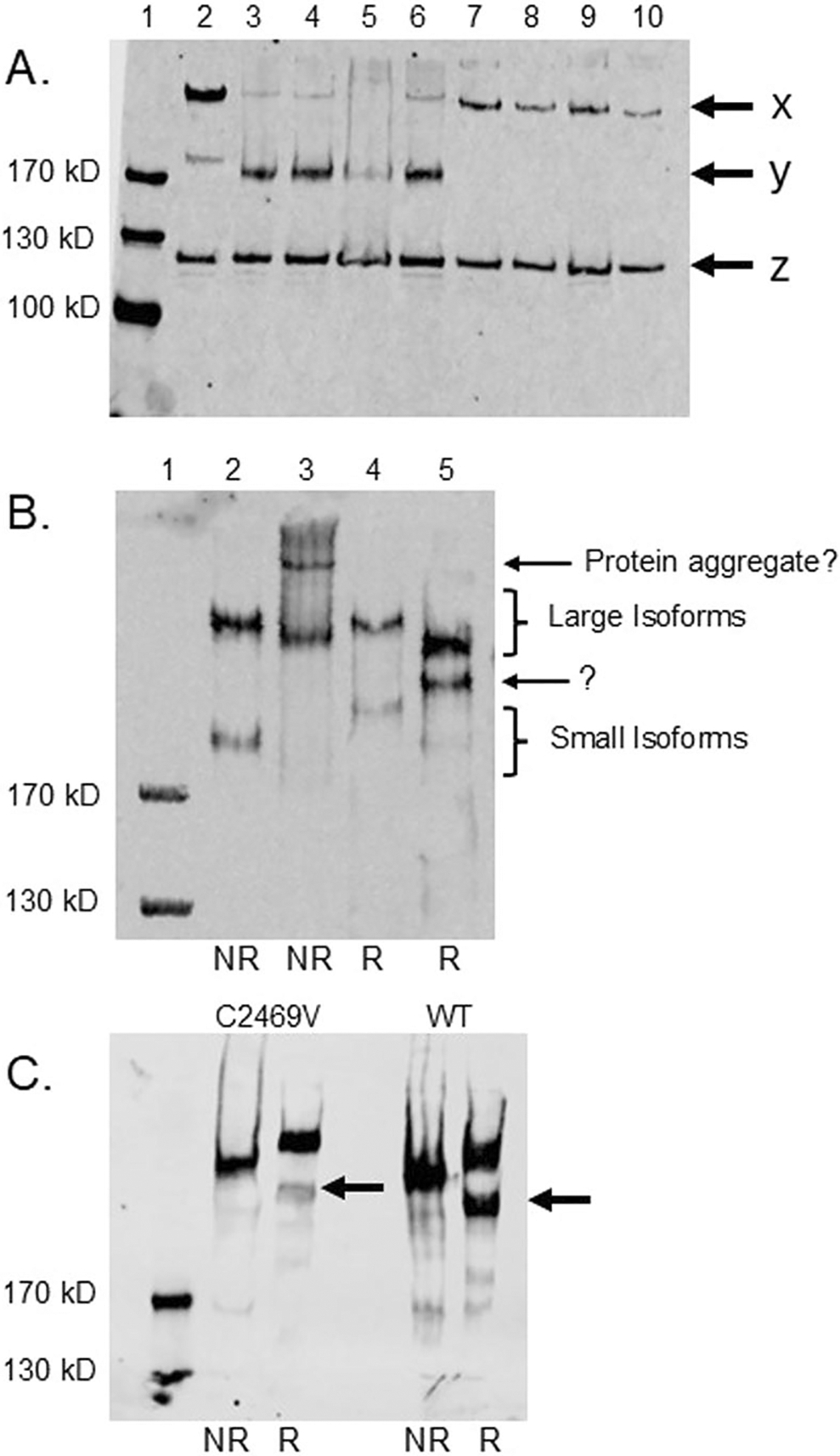
Generation of stable cell lines and protein expression characteristics. FlpIn HEK293 cells stably expressing either rat 300HARE or rat 175HARE were generated similarly to human cell lines [19, 34]. (A) Twenty micrograms of cell lysate protein from four different 175HARE and 300HARE clones were separated by 5% SDS-PAGE and detected by anti-V5 antibody which detects the epitope tag on the C-terminus of the protein. Arrow (x) indicates the large isoform and arrow (y) indicates the smaller isoform. (A) monoclonal antibody against vinculin was used as a load control, arrow (z). Lane 1: Protein ladder, Lane 2: Human Stab2/HARE, Lanes 3–6: rat 175HARE, Lanes 7–10: rat 300HARE (B). Comparison of non-reduced (NR) and reduced (R) cell lysates from human and rat HARE separated by 5% SDS-PAGE. Lane 1: Protein ladder, Lane 2: non-reduced Human 315HARE, Lane 3: non-reduced rat 300HARE, Lane 4: reduced human 315HARE, and Lane 5: reduced rat 300HARE. The arrow with question mark indicates a consistent prominent band unknown HARE (C). The C2469V was generated and transiently transfected in FlpIn HEK293 cells and compared with WT rat 300HAREBoth nonreduced and reduced cell lysates were separated by 5% SDS-PAGE. The arrows indicate confirm the existence of a secondary band of unknown HARE only present in reduction which is less prominent in the C2469V mutant.

The large (300 kDa) and small (175 kDa) rat HARE isoforms were first cloned from rat spleen tissue using the Gibson Assembly method in which the cDNA was inserted into pcDNA5/FRT/V5/6xHIS vector (Life Tech, CA) and stable cell lines were made that expressed the receptor (see methods). Expression levels were assessed by Western blot of four clones of each isoform and these were compared to the stable human HARE/Stab2 line that we characterized previously which was clone 30 [19]. In Figure 1A lane 2 of the blot, cell lysate from the stable clone expressing human large 315HARE/Stab2 consistently produces the smaller 190HARE receptor. The small rat isoforms expresses similarly to the human 190HARE isoform when expressed independently as demonstrated in lanes 3–6 of Figure 1A, arrow y [34]. The rat 300HARE/Stab2 was expressed consistently as expected (arrow x), but we did not initially observe the generation of the smaller isoform in these cell lysates from several stable clones (Figure 1A, lanes 7–10). All cell lysate protein loads were similar as indicated by the vinculin load control (arrow z). When the 300HARE cell lysates were concentrated to a higher level, we began to observe the smaller 175HARE isoform which became more prominent when the protein was reduced (Figure 1B, lane 5). Since the generation of the small isoform is a result of proteolytic cleavage from the large isoform, lower levels of the isoform may be the result of inefficient processing of a rat protein expressed in a human cell line. The occurrence of two major bands upon reduction of 300HARE was surprising, but not unprecedented (Figure 1B, lane 5). Reduction and deglycosylation of the native 300HARE from primary rat liver sinusoidal endothelial cells resulted in the appearance of 260, 230, and 97 kDa fragments in another laboratory [13]. Apparently, most receptor processing features of the recombinant 300HARE persisted in the stable HEK293 cell line.

One of the distinctions between human and rodent HARE/Stab2 is the presence of a cysteine residue in the transmembrane domain at position 2469 (Figure S1). This cysteine probably does not form disulfide bonds with other cysteines and may be found in the original sulfhydryl form or oxidized to various forms [36]. When this cysteine was mutated to a valine, the smaller of the 300HARE fragments (indicated by the arrows) in the reduced protein (R) when compared with the nonreduced protein (NR) began to diminish, suggesting that this cysteine is involved but not critical in the processing of the receptor (Figure 1C).

**FIGURE 2 |:**
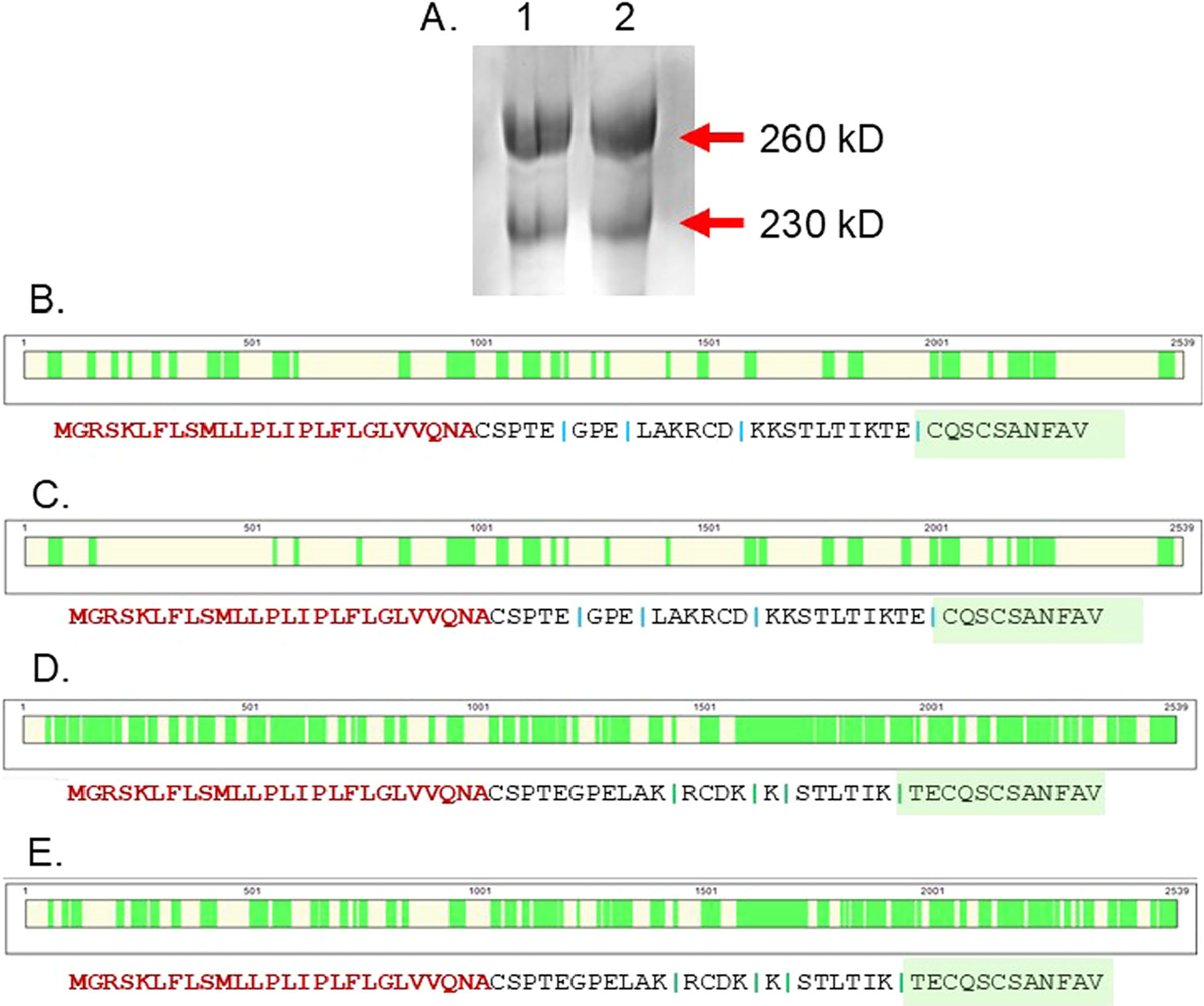
Mass spectrometry coverage of the reduced 300HARE. Antibody 30 specific for rat HARE was used to immunopreciptate r300HARE from a cell lysate. A. Rat 300HARE was reduced and bands of 260 and 230 kDa was separated by SDS-PAGE in duplicate (lanes 1 & 2). The Coomassie-stained proteins were cut from the gel and digested with V8 (Figure 2B and C) or with Trypsin (Figure 2D and 2E). The percentage coverage for these each protein in B–E was 31%, 24.5%, 68.1%, and 58.6%, respectively. The N-terminus of each protein is written in red lettering was not detected in any of the peptides and is the signal sequence that is cleaved off in mature receptors. The green boxed text represents the first peptide detected from the N-terminus.

Since reduction of the rat 300HARE yielded protein fragments that were approximately 260 and 230 kDa in size, we assumed that this was the result of proteolytic cleavage as seen in several previous publications [13, 14]. The 300HARE from a cell lysate was immunoprecipitated with mAb30 conjugated to a sepharose resin, reduced, and alkylated. The purified and reduced 300HARE was separated on a 20 cm 5% SDS-PAGE and Coomassie stained (Figure 2A, lanes 1 & 2 which are duplicate). Bands from each protein mass were cut from the gel and combined and analyzed by mass spectrometry using peptides generated from both trypsin and V8 digests (Figure 2B–E). The results from this analysis verified that each band is a copy of the 300HARE according to the peptide sequences. The mass spectrometry peptide coverage was sufficient to confirm that the difference in size of the reduced protein fragments is not a result of proteolytic processing, but of some other factor that remains unknown at this time (Figures S2, S3). This is quite surprising since this is not observed with the human 315HARE protein [19]. With the appearance of a second lower molecular weight band upon reduction of the rat receptor, we had assumed that this was proteolytic cleavage of the large isoform during processing between the Golgi and secretion to the plasma membrane and all these distinct parts of the receptor were held together by disulfide bonds that were established during folding/processing in the ER. At this time, we cannot explain why this form of the full-length protein migrates at a lower molecular mass after reduction, except that maybe there is a second stabilized physical conformational change that allows it to migrate faster through the acrylamide matrix. What if the protein was not fully reduced? This was tested by the addition of increasing amounts of dithiothreitol (DTT) with constant 10 mM iodoacetamide. Our results indicate that our lowest amount (1 mM) of DTT fully reduced 300HARE as the band intensity of 260/230 did not increase with increasing DTT. The Laemmli solution that the reduction reaction occurred in also changed from blue to yellow (bromophenol blue) as more DTT was added verifying that reduction was complete (Figure S4). Partial reduction is not why there are two sizes of the same protein present. We can only verify that both bands in Figure 2A are the identical proteins.

**FIGURE 3 |:**
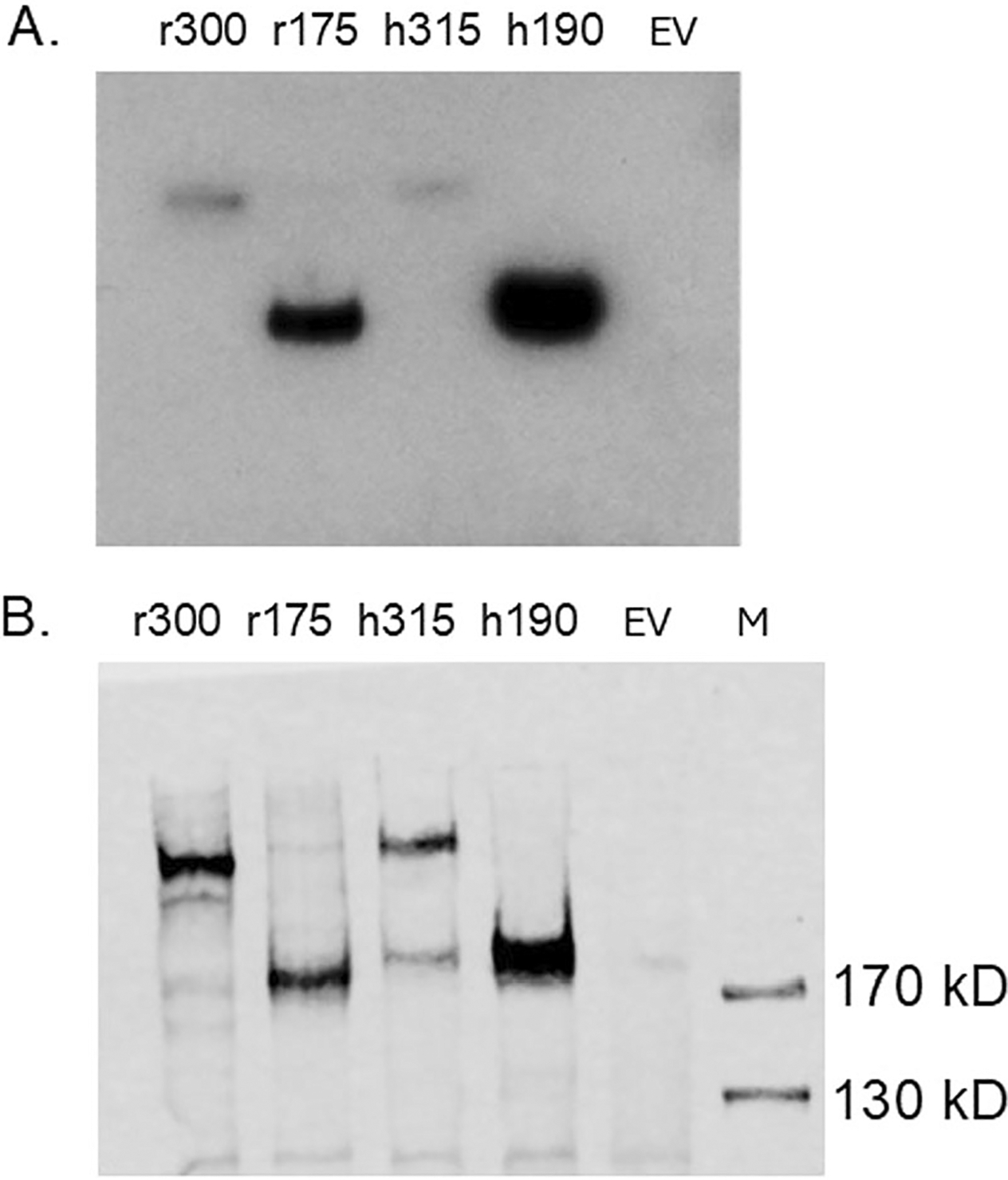
Ligand and Western blots for HA binding. (A) Cell lysates (40 μg) from human, rat HARE and empty vector (EV) control were separated by 5% SDS-PAGE, blotted, and exposed to 1 μg/mL ^125^I-HA for 1 h at room temp. The blot was dried, wrapped in cellophane, and exposed to film at −80°C. (B) The same blot was then rehydrated in blocking buffer (TBS, 0.05% tween-20, 1 mg/mL BSA) followed by standard Western blot procedures using anti-V5 to detect the HARE proteins.

**FIGURE 4 |:**
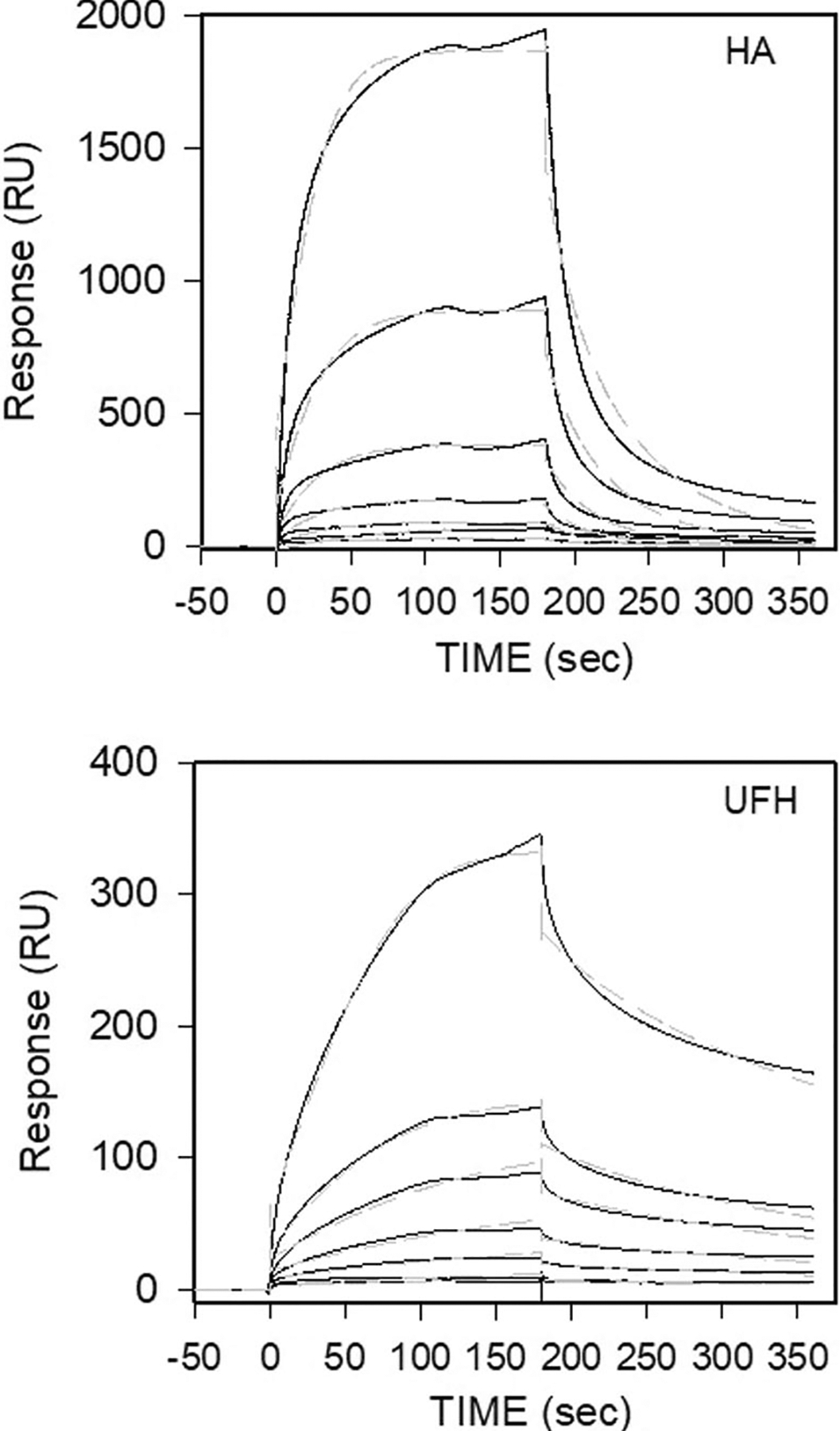
Surface Plasmon Resonance of sr175HARE with hyaluronan and heparin. (A) Biotinylated HA and (B) biotinylated UFH was attached to a Streptavidin (SA) biosensor chip (0.1 mg/mL at flow rate 10 μL/min) and association and dissociation rates of 3.9, 7.8, 15.6, 62.5, 125, 250 nM sr175HARE analyte were measured under flow. Black solid lines are collected data from a Biacore T200 instrument, and the dashed gray lines are fitted curves derived from the T200 evaluation software (version 3.2). The artwork is plotted by SigmaPlot version 11.2.

To verify that the 300HARE is active against a known ligand, we incubated human 315HARE/190HARE and rat 300HARE/175HARE cell lysates that were separated by SDS-PAGE and then blotted to nitrocellulose with iodinated hyaluronan (^125^I-HA) in Figure 3A. When expressed independently, the smaller isoforms have much more robust expression in the cells in terms of quantity of receptor. After exposure to film, the same nitrocellulose sheet used for radiolabeled ligand blot was used in a Western Blot to probe for each HARE isoform via the anti-V5 antibody to make a qualitative assessment of protein load relative to HA binding (Figure 3B). Due to the high electronegative characteristics of heparin polymers, the ligand blot method does not work well and suffers from high background issues. To verify UFH binding and to obtain kinetic on/off rates for both HA and UFH, we used surface plasmon resonance to determine overall K_D_ values. The small rat isoform was used in all experiments and contains all the binding sites for both HA and UFH. Biotinylated HA (Figure 4A) and UFH (Figure 4B) were injected at 0.1 mg/mL at a flow rate of 10 μl/min and immobilized on Streptavidin (SA) chips and concentrations of secreted rat 175HARE (sr175) at concentrations ranging from 3.9 to 250 nM were allowed to bind and dissociate. The solid black lines are the kinetic data obtained from the sensorgram and the dotted gray lines are the fitting curves using a model from T200 Evaluation software (v. 3.2). Association and dissociation rates with overall K_D_ values are summarized in Table 1. Two versions of biotinylated heparin (one from the Harris lab [UFH] and one from the Zhang lab [Heparin]) were used for this experiment to ensure that the UFH data was correct. The K_D_ value of 31.5–36 nM for UFH for the rodent protein is within the accepted ranges for the human 190HARE (s190). The HA binding data for 175HARE is quite different from the 190HARE.

Previously, we measured the 190HARE-HA binding to be a value of K_D_ = 5–23 nM [20] which contrasts to our current 175HARE-HA values at 212 nM. This may be due to the high specificity/affinity or a steep incline observed for ka and a very steep kd when ligand washes off rapidly. Rodents tend to have high HA concentrations in circulation and from what has been observed, the 175HARE (and likely 300HARE) are specific for HA but the high affinity should not be mistaken for high binding strength. In lessons learned from the 190HARE, the HA affinity is highly specific, but the strength of the binding interaction is weak compared to other hyalectins such as CD44, TSG-6, and Versican Link [37]. The HA binding strength appears to be lower in rat Stab2/HARE than in human Stab2/HARE which is quite logical for a recycling receptor. The Stab2/HARE must have high affinity for HA, but also releases it easily in the endosome as the empty receptor recycles back to the cell surface. This may also be the reason for why rat Stab2/HARE outperforms human Stab2/HARE in HA endocytosis and not UFH endocytosis as outlined below.

**FIGURE 5 |:**
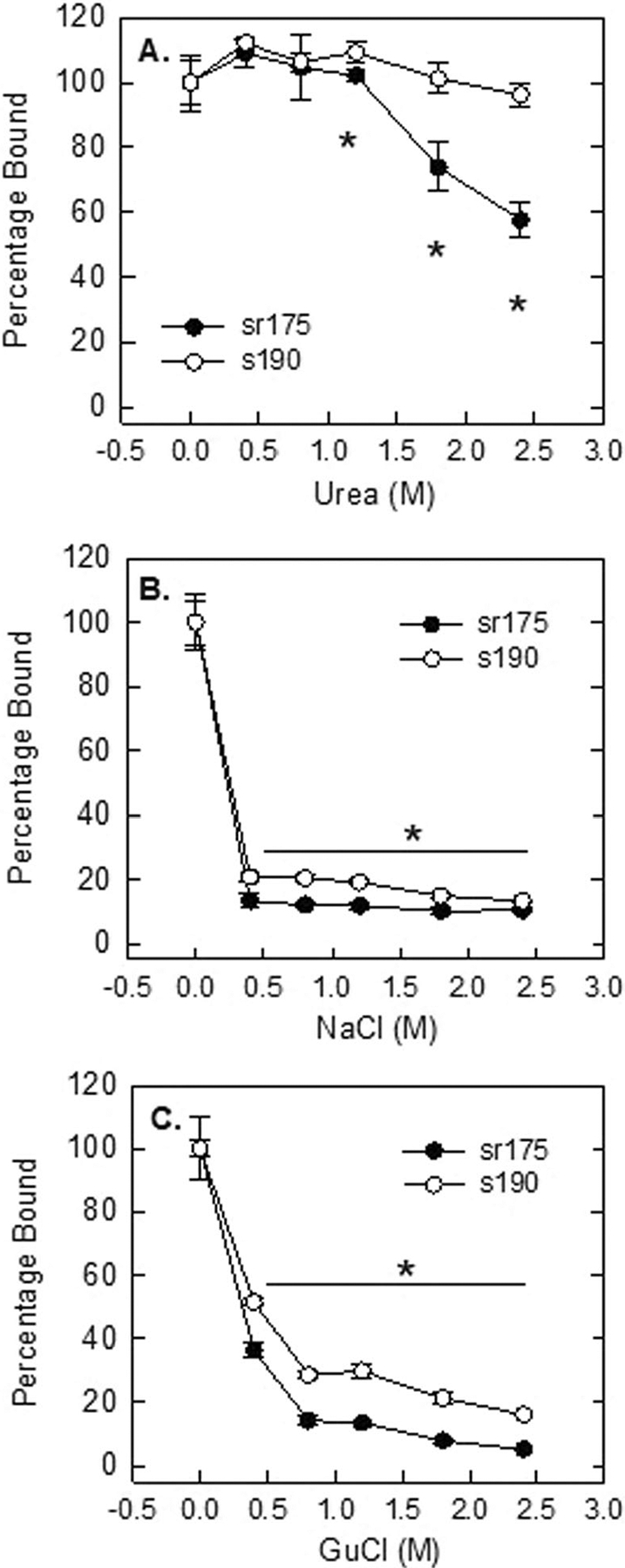
Direct binding assay. Purified human s190 and rat sr175 proteins were bound to polysorp wells and incubated with 200 nM biotinylated UFH in the presence of 0, 0.4, 0.8, 1.2, 1.8, 2.4 M (A) urea, (B) NaCl, and (C) GuCl. For each concentration, *n* = 4 ± s.d., *p < 0.001.

To further investigate the nature of heparin binding with both secreted human and rat Stab2/HARE, we used a direct binding assay in a static plate format to determine the effects of salts on this interaction. We used a range of 0.4–2.4 M urea, to disrupt hydrogen bonding, sodium chloride to disrupt ionic bonding, and guanidinium chloride to disrupt protein folding. The s190 and sr175 proteins were bound to the sides of the polysorp wells and incubated with biotinylated heparin after blocking with BSA. Bound heparin was detected by ^125^I-streptavidin and assessed by a gamma counter. All values were normalized to the control that did not contain any additional salts. Both s190 and sr175 do not have an obvious linear sequence of alkaline (positively charged) amino acids that are commonly found in many heparin binding proteins [38, 39]. The addition of urea did not significantly disrupt binding for the s190, however, the sr175 was significantly disrupted past 1.2 M urea (Figure 5A). The sulfates of the heparin polymer are thought to be the primary residues that interact with amino acids forming salt or ionic bridges. As predicted, increasing sodium chloride disrupted heparin binding significantly, but more so in the rat than in the human protein (Figure 5B). Since these proteins do not have linear or clustered alkaline amino acids, it is thought that distal alkaline residues come together to form a binding cleft or pocket. The addition of increasing guanidinium chloride significantly disrupted binding in both proteins from 0.4 M, however, the sr175 was affected more severely (Figure 5C). To ensure that the highest salt concentrations did not affect s190 or sr175 binding to plastic, we performed a control assay in which bound protein was detected by anti-V5 antibody. This antibody was chosen due to the V5 tag that does not require folding. Primary antibody was then detected by secondary linked with alkaline phosphatase and measured by a plate reader for color development. There was no significant loss of s190 and a slight loss of sr175 with 2.4 M GuCl (Figure S5). Without knowing the structure of these proteins, these data suggest that the sr175 is more sensitive to increasing salt concentrations that may alter folding more readily impacting the heparin binding site than what we observe with the s190.

**FIGURE 6 |:**
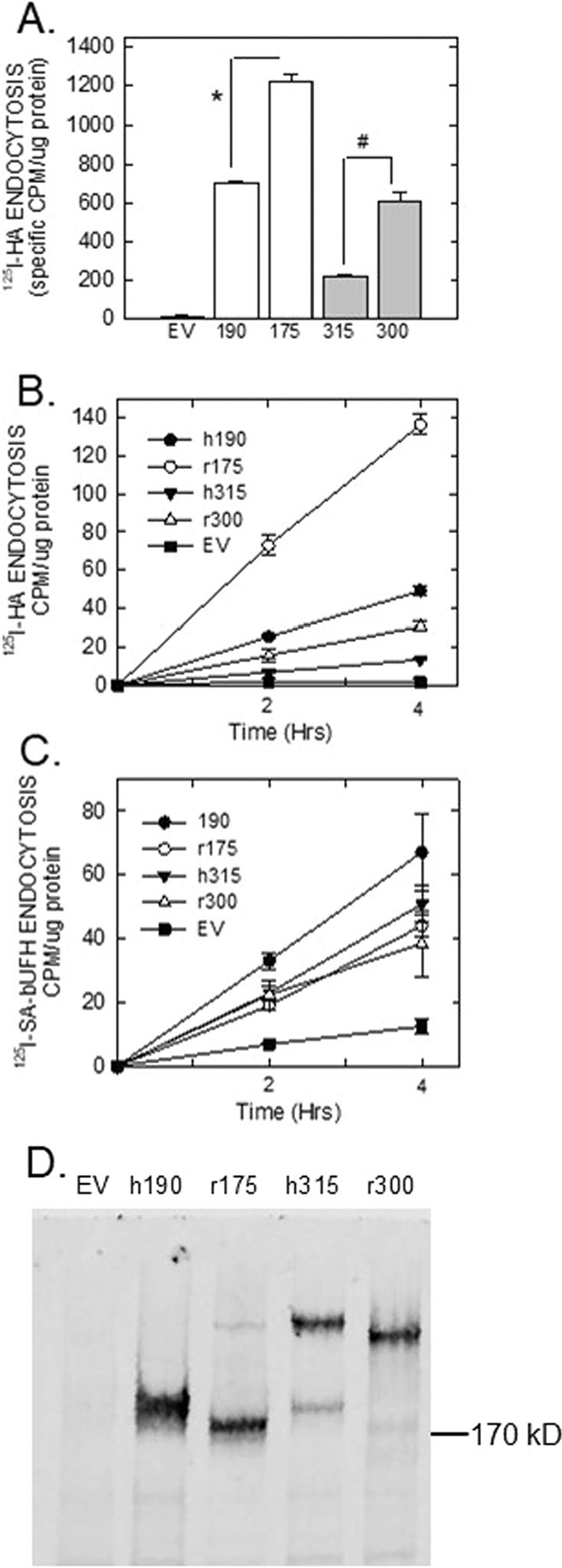
Endocytosis of HA by human and rat HARE/Stab2. (A) EV (empty vector) 315HARE, 190HARE and four clones each of 300HARE and 175HARE were plated in 24-well tissue culture plates and allowed to grow up to about 80% confluency. Biotinylated HA or UFH were prepared as described in methods and 200 nM of biotinylated GAG was conjugated with 2.5 mg/mL ^125^I-SA diluted in plain DMEM for a working volume of 0.3 mL per well in each plate. ^125^I-SA alone was used for background control. Cells were allowed to internalize radiolabeled ligands for 4 h, washed, lysed in 0.3 M NaOH and radioactivity was assessed by a gamma counter and total lysate protein was measured using the Bradford method. Individual clones identified from part A were incubated with either radiolabeled SA conjugated with (B) HA or (C) UFH and cells harvested at 2 and 4 h. (D) Cell lysates from the cell lines used on (C) were collected and 20 mg of total protein was separated by 5% SDS-PAGE and HARE was detected by anti-V5. The top band of the marker for 170 kD is equivalent to the marked point of this blot. For each time point, *n* = 4 (mean ± SD for human 315HARE and 190HARE and all cell lines in [B] and [C]). For part A, 300HARE and 175HARE were *n* = 4 (mean ± SEM). *p < 0.05.

Following in vitro binding by SPR and direct binding assay, we tested binding and calculated specificity of both ^125^I-SA-bHA and ^125^I-SA-bUFH with cell lines expressing human 315HARE and 190HARE as controls along with cell lines expressing the rat 300HARE and 175HARE. Relative amounts of HA endocytosis were typical with the rat lines internalizing significantly more than their human counterparts (Table 2). Furthermore, surface binding for HA was above 50% for all lines except the 300HARE. This may be due to low expression of the receptor at the cell surface which does not have much impact on internalization rates. Other investigators examining the human Stabilin-1 elegantly demonstrated that high endocytic capacity does not necessarily rely on high surface expression, but rather turnover rate of the receptor from the surface to endosomes and recycling back to the surface [40]. For UFH binding, we just examined the 315HARE and 300HARE and determined that the 315HARE had half the specificity at the surface in contrast to total binding which includes receptors that are within endosomes. The 300HARE had lower surface specificity, but the internal levels of binding were the approximately the same which was confirmed by Western blot for total cellular expression (Table 3, Figure 6D).

Next, we assessed the ability of cell lines stably expressing rat HARE/Stab2 for endocytosis of HA and UFH compared with characterized human Stab2/HARE. HA and UFH endocytosis activity was measured for each group of cell lines as shown in Figure 1. Four cell lines from each rat HARE group were averaged against the human 315HARE clone 30 and 190HARE clone 9 that were characterized previously [19, 34]. Using standard deviations for experimental replicates in the human lines and the standard error for multiple rat lines, it was determined that both rat isoforms exhibit robust HA endocytosis which exceeded the human isoforms (Figure 6A) at the 4 h time point. The optimal cell lines were also measured individually for both HA and unfractionated heparin (UFH) endocytosis for a time course of 4 h (Figure 6B, C, respectively). Although HA endocytosis with the rat HARE is consistently robust and exceeded the human HARE measurements, this was tempered when viewing heparin endocytosis in which the all the cell lines were approximately similar in their internalization rates (Figure 6C). All data were normalized to protein levels and receptor expression and a representative blot of the protein expression in 20 μg cell lysate protein is shown in Figure 6D. It may be worth noting that the smaller isoform may be faintly observed in the r300 lane and runs at the same rate as the 175HARE in 5% SDS-PAGE (faint band near the 170 kD line) (Table 3).

**FIGURE 7 |:**
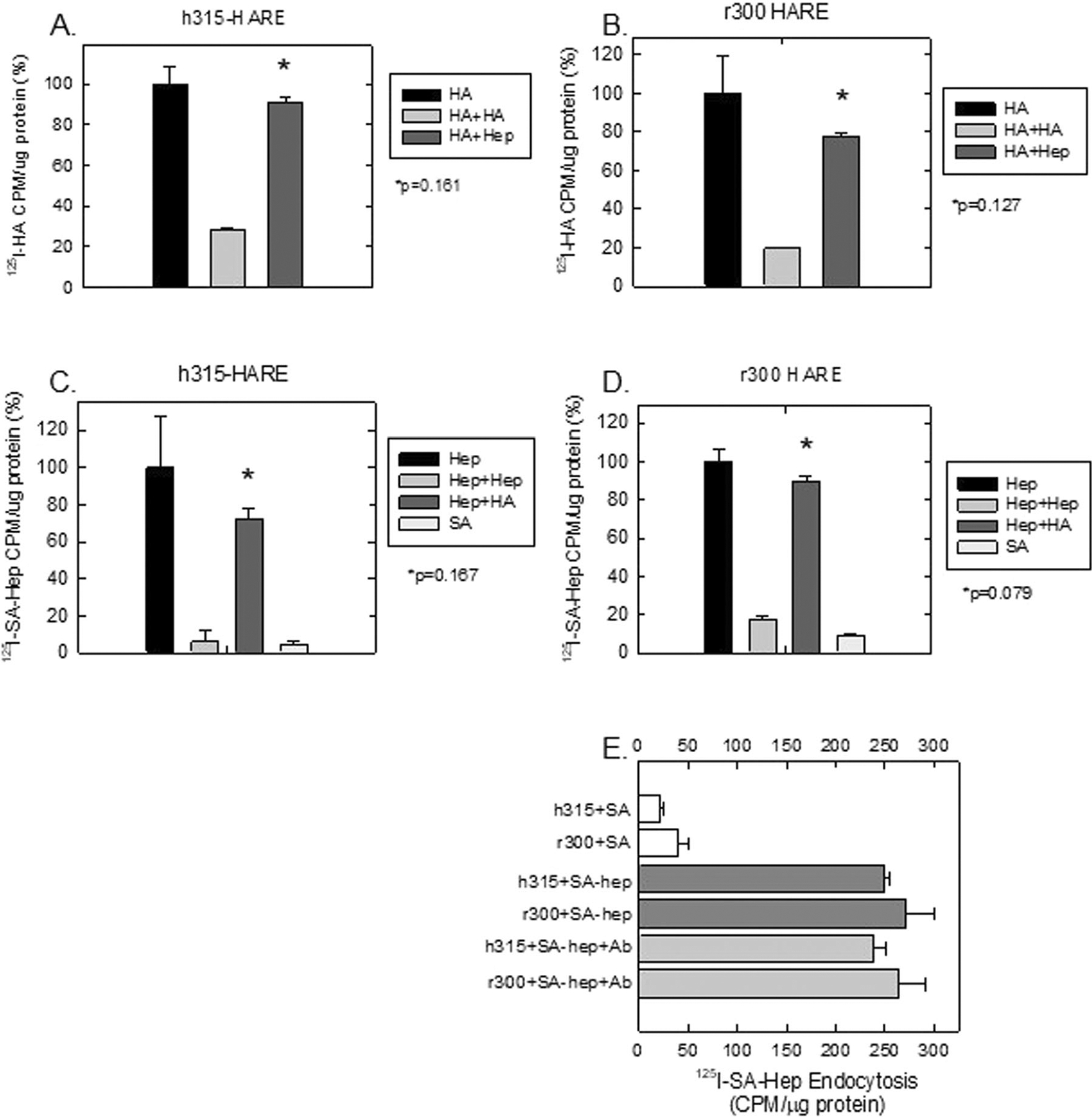
Cross-competition of ligands for 315HARE and 300HARE. (A) and (B): Cell lines were plated in 24-well plates and incubated with 1 μg/mL ^125^I-HA alone or with 100-fold excess HA or UFH. (C and D): Cell lines in 24-well plates were incubated with ^125^I-SA-bUFH alone or with 100 μg/mL UFH or HA as competing ligands. ^125^I-SA alone was used to determine background radiation levels and all incubations were for 2 h. (E) Cell lines were plated in 24-well plates with either 2.5 mg/mL ^125^I-SA alone or ^125^I-SA-bUFH or ^125^I-SA-bUFH + Antibody 174 for 2 h.

We had previously established in human HARE/Stab2 that heparin and HA bound to the 190HARE and 315HARE receptors in a mutually exclusive manner [26]. The notion that Stab2/HARE may bind multiple ligands at the same time without cross-competition has been missed previously [27]. In Figure 7A, we repeated the 315HARE experiment using ^125^I-labeled HA with 100-fold excess unlabeled HA or UFH similar to previously published results [35]. These experimental conditions were repeated with the rat 300HARE (Figure 7B). The converse remained true with 125I-labeled streptavidin bound with biotinylated UFH and incubated with 100-fold excess UFH or HA in 315HARE (Figure 7C) and 300HARE (Figure 7D). Since Streptavidin contained the radiolabel, we also incubated cells with 125I-Streptavidin alone to assess background radiation associated with endocytosis within the experiment. Monoclonal antibody 174 reduces HA endocytosis by up to 50% and, presumably, binds to the LINK domain of HARE/Stab2 [41]. To confirm that the Link domain of both human and rat HARE is not associated with UFH binding, we incubated cells alone with ^125^I-SA, ^125^I-SA-bhep + IgG, and ^125^I-SA-bhep + Ab174. We observed no difference between the control IgG and mAb174 for UFH binding and endocytosis, giving further evidence that heparin does not bind to the LINK domain and does not interfere with HA binding (Figure 7E).

**FIGURE 8 |:**
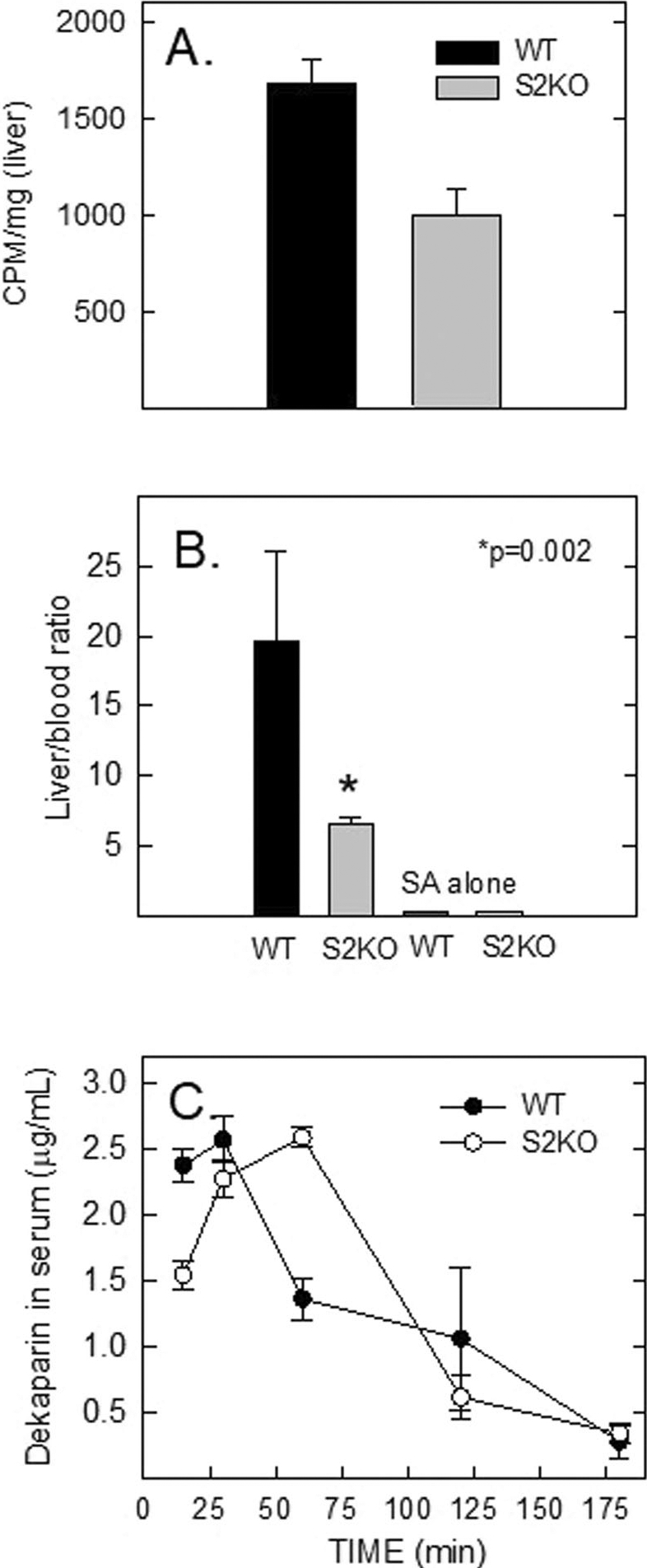
Heparin accumulation in WT and HARE/Stab2KO mice. (A) Both WT and Stab2KO mice were injected with radiolabeled UFH which was allowed to circulate for 20 min. The mice were anesthetized with isoflurane, allowed to bleed out, and liver tissue was collected and evaluated for radiolabel accumulation. Mean ± SD, *n* = 6, *p* = 0.007. (B) The liver to blood ratio of UFH is compared in WT (black) and Stab2KO (gray) mice. Mean ± SD, *p = 0.002, *n* = 6. ^125^I-SA alone was also evaluated in the same manner. (C) A dose of 0.6 mg/Kg Dekaparin was injected subcutaneously in WT and Stab2KO mice and retro-orbital bleeds were performed at the indicated times. The amount of Dekaparin was measured in plasma by mass spectrometry. *n* = 5 mice per time point. Mean ± SD, *p ≤ 0.001 at 15 and 60 min time points.

The Stabilin-2 knockout (Stab2KO) mouse has previously been characterized in Hirose et al. [29]. This same mouse strain was obtained by the Harris laboratory, and we performed some initial characterization of liver sinusoidal endothelial cells (LSECs) with UFH using in vitro experiments. We found that heparin internalization by cell culture methods was barely significant compared to WT cells [42]. In these in vivo experiments, we discovered that there is a clear distinction of UFH sequestration in the livers of WT mice compared with Stab2KO mice. Mice were injected with a mixture of 200 nM ^125^I-SA-bUFH by intravenous injection and allowed to circulate for 20 min. Mice were anesthetized by isoflurane and blood and liver tissue were collected and evaluated (Figure 8A). Recalculating the numbers in terms of liver to blood ratio of radioactivity/weight also revealed a significant difference between WT and Stab2KO mice (Figure 8B). We also injected mice with ^125^I-SA alone which demonstrated that there was no difference in liver and blood content of radioactivity which strongly suggests that the LSECs are not taking up any radioactive streptavidin (Figure 8B, small bars on the right).

Since UFH is a heterogenous polymer with different binding activities, we injected mice subcutaneously with Dekaparin which is a homogenous dodecamer containing one 3-*O* sulfate that is active against Factor X, Stab1 and Stab2 [30]. The 3-*O* sulfated dodecamer has five times the affinity as the non-3-*O* sulfated dodecamer despite the other 16 sulfate groups that are present [43]. It is presumed that charge and orientation of the sulfates are important for heparin-Stab2 binding with the lone 3-*O* sulfate group having the most significant impact (Figure S6). After subcutaneous injection, we collected blood at specific time points between 15 and 180 min from WT and Stab2KO mice and the amount of Dekaparin was evaluated by LC-MS/MS (Figure 8C). The results show that the Stab2KO mice have a delay in Dekaparin metabolism in which there is lower liver sequestration and more remaining in blood and filtered out by the kidneys. By 180 min, both WT and Stab2KO cleared Dekaparin from the plasma. Clearly, the Stab2KO mouse has other receptors that bind with Dekaparin. A very likely candidate is Stab1 which is another heparin binding receptor [43] as well as other possible receptors such as SR-A [44] and low-density lipoprotein [45]. Clearly, the omission of Stab2/HARE delays metabolism of Dekaparin and increases plasma accumulation of the drug, exerting more pressure on kidney filtration.

## 4 | Conclusion

This is the first manuscript for the characterization of the rat recombinant full-length Stab2/HARE receptor. We have confirmed that the full-length receptor is expressed as two isoforms at 300 kDa and 175 kDa similar to the human 315 kDa and 190 kDa. However, expression of the smaller isoform in human HEK293 cells is significantly smaller than the human homolog. Reduction of the 300 kDa isoform results in the appearance of two bands, one at 260 kDa and the other at about 230 kDa. These were observed previously and reported by Zhou et al. [13]. Previously, it was assumed that the 300HARE was a multimeric protein that was held together by disulfide bridges. Our results confirmed that each distinct band of 260 kDa and 230 kDa separated by SDS-PAGE identified as the r300HARE, it is presumed that two stabilized forms of the reduced protein allow one to travel faster through acrylamide gels. More experimentation needs to be performed to elucidate these two physical states of this enigmatic receptor.

Both 300HARE and 175HARE bind to HA and heparin. HA affinity for the rat 175HARE is not as high as the human 190HARE, however, the specificity meets the threshold for stable binding and internalization. The high dissociation rate allows for better efficiency for cargo release within the cell that may drive higher rat receptor cycling as both human and rat receptors are constitutively active [34, 46]. Since HA binds to the Link domain of Stab2/HARE and both isoforms contain only one Link domain, we can reasonably suggest that HA binding and kinetics are the same for both isoforms. Both rat and human small isoforms bound UFH with the same affinity, though the kinetics were increased in the rat cell lines. Rat Stab2/HARE is more sensitive to increasing salt and loses heparin binding capability in contrast to the human receptor suggesting that 3D conformation may be more dynamic for unfolding and adjusting to ligand binding.

## Supplementary Material

1

## Figures and Tables

**TABLE 1 |:** Kinetic data of proteins binding with HA/heparin.

	K (M^−1^ S^−1^)	K_d_ (S^−1^)	K_D_ (M)
HA	1.02 × 10^6^, (±4.7 × 10^4^)^a^	2.16 × 10^−1^, (±1.0 × 10^−2^)^a^	2.12 ×10^−7^
UFH	2.33 × 10^5^, (±2.7 × 10^3^)^a^	7.34 × 10^−3^, (±5.4 × 10^−5^)^a^	3.15 × 10^−8^
Heparin	1.88 × 10^5^, (±1.8 × 10^3^)^a^	6.76 × 10^−3^, (±4.1 × 10^−5^)^a^	3.6 × 10^−8^

a The data with the (±) in parentheses represent the standard deviation (SD) obtained from the global fitting of five injections.

**TABLE 2 |:** HA endocytosis, binding and specificity to Stab2/HARE.

Cell line	Endocytosis (CPM/μg protein)	Surface sinding (CPM/μg protein)	Total Binding (CPM/μg protein)	Endocytosis specificity (%)	Surface Binding Specificity (%)	Total binding specificity (%)
190HARE	296.9	31.85	635.8	97.0	57.6	96.0
315HARE	107.0	16.9	366.0	91.0	82.8	96.3
175HARE	497.7	43.7	95.1	97.3	84.3	89.3
300HARE	118.5	15.3	50.1	88.2	11.7	22.1

**TABLE 3 |:** UFH endocytosis, binding and specificity to Stab2/HARE.

Cell line	Endocytosis (CPM/μg protein)	Surface binding (CPM/μg protein)	Total binding (CPM/μg protein)	Endocytosis specificity (%)	Surface binding specificity (%)	Total binding specificity (%)
315HARE	91.1	24.2	55.6	81.4	35.7	65.7
300HARE	63.0	21.5	69.7	86.0	11.2	60.6

## Data Availability

The data that support the findings of this study are available on request from the corresponding author. The data are not publicly available due to privacy or ethical restrictions.
