# Supplementary material for: Characterization of Heparin Interactions With Recombinant Rodent Stabilin-2/Hyaluronic Acid Receptor for Endocytosis (HARE)
Source: Proteoglycan Res. Author manuscript; Available in PMC 2025 Aug 29. (PMC12393792; doi:10.1002/pgr2.70027)
Supplement: 1 [file NIHMS2081180-supplement-1.pdf]

Characterization of Heparin Interactions with Recombinant Rodent Stabilin-2/Hyaluronic Acid Receptor for Endocytosis (HARE)

[1]Reed A. Rohr, [1]Evan A. Schroder, [1]Joseph D. Staab, [1]William P. Singh, [1]Callan J. Schroder, [1]Grant D. Hatcher, [1]Joshua T. McWilliams, [3,4]Jiyuan Yang, [1]Abby E. Bopp, [1]Linda B. Fatumoju, [2]Zhangjie Wang, [4]Jonathan S. Dordick, [4]Robert J. Linhardt, [4]Fuming Zhang, [2]Jian Liu, [1]Edward N. Harris‡

[1]University of Nebraska, Lincoln NE 68588 USA, [2]University of North Carolina Chapel Hill, Chapel Hill, NC 27009, [3]The Key Laboratory of Molecular Microbiology and Technology, Ministry of Education, College of Life Sciences, Nankai University, Tianjin 300071, P.R. China, [4]Department of Chemical and Biological Engineering, Rensselaer Polytechnic Institute, Troy, NY, 12180, USA

Supplemental figures

```
Human LTHTGLGAGIFFAIILVTGAVALAAYSYFRINRRTIGFQHFESEEDINVAALGKQQPENISNPLYESTTSAPPEPSYDPFTDSEERQLEGNDPLRTL
Mouse AAHSGLGTGIFCAVVLVTGAIALAAYSYFRLNQRTTGFRRFESEDDIDALAFGKQQPESITNPLYETSTPAAPEPSCDPFTDSGERELENSDPLGALRS
Rat   AAHSGLGTGIFCAVVLVTGAIALAAYSYFRLKQRTTGFQRFESEEDIDVLAFGKQQPKNIANPLYETSAPAPPESSCDPFTDPGEQDLEDSDPLGALRS
        * *** *** *  ***** *********   ** **  **** **   * *****  * *****    * ** * *****  *  **  ***  *
```

Fig. S1: Alignment of the transmembrane (red) and cytoplasmic (green) regions of human, mouse, and rat Stab2/HARE. The blue arrow points to a phenylalanine for the human protein which are cysteines in the rodents.

Trypsin digests

260 kDa

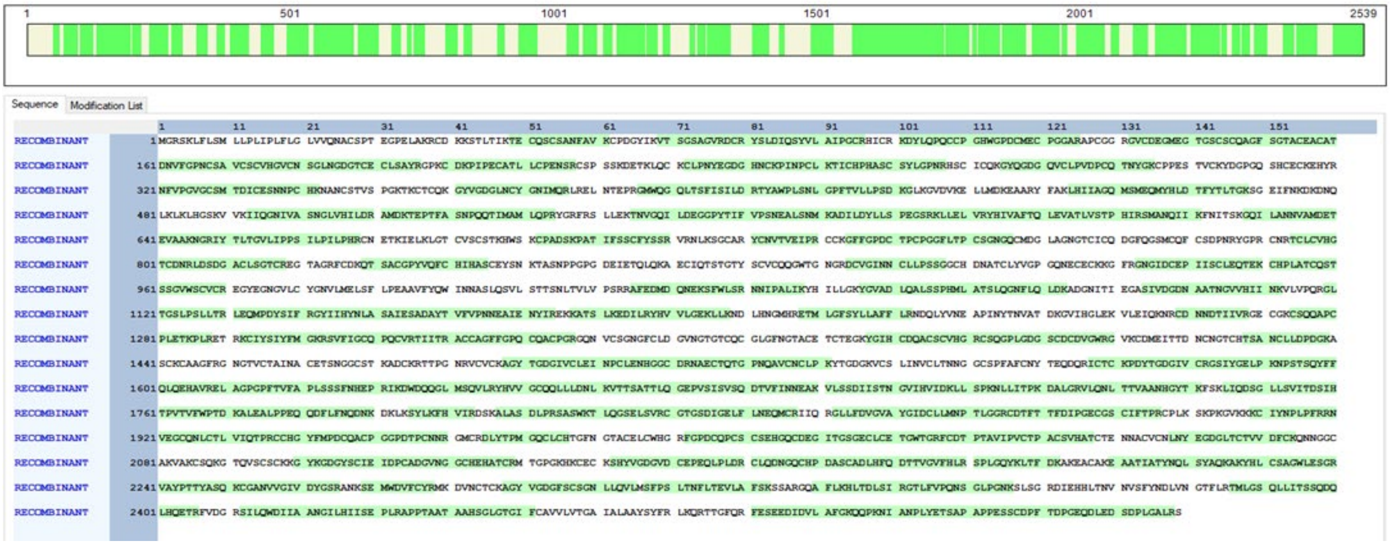

230 kDa

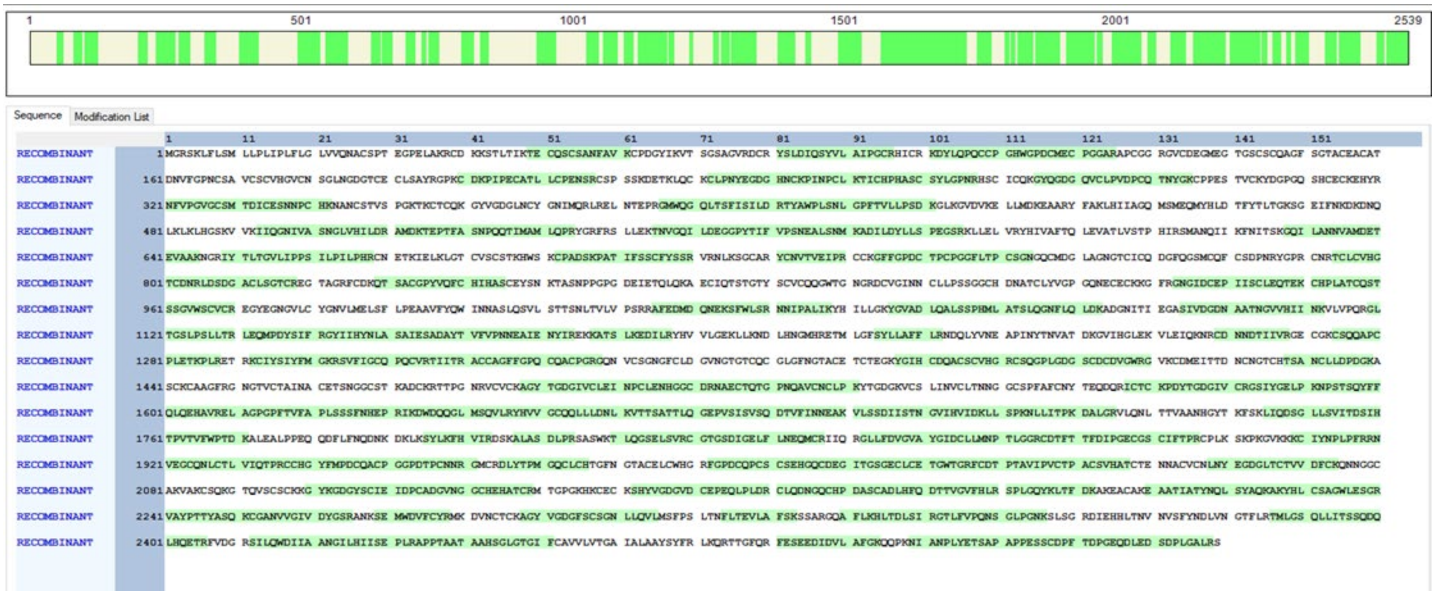

Fig. S2: Mass spectrometry coverage of the protein bands extracted from SDS-PAGE. Trypsin digest of the 260 and 230 kDa bands yielded a coverage of 68.1% and 58.6%, respectively. Sequences outlined in green represent the peptides that were identified by mass spectrometry. The bar just above the protein sequence is a representative coverage map of identified (green) protein.

V8 digests

260 kDa

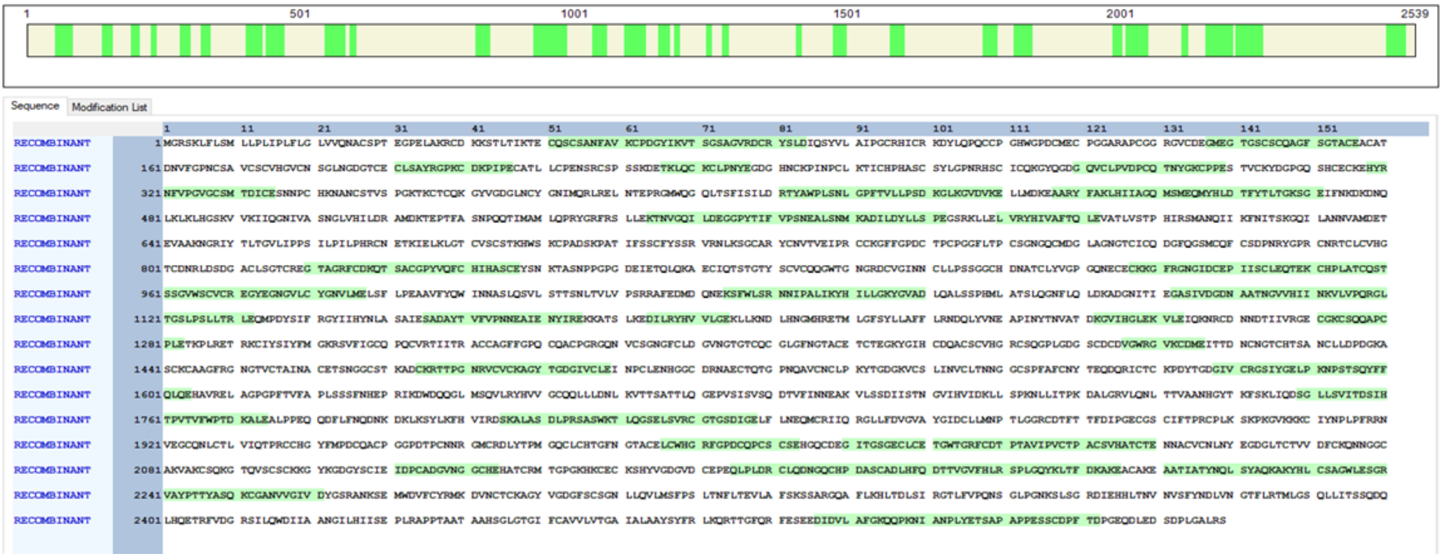

230 kDa

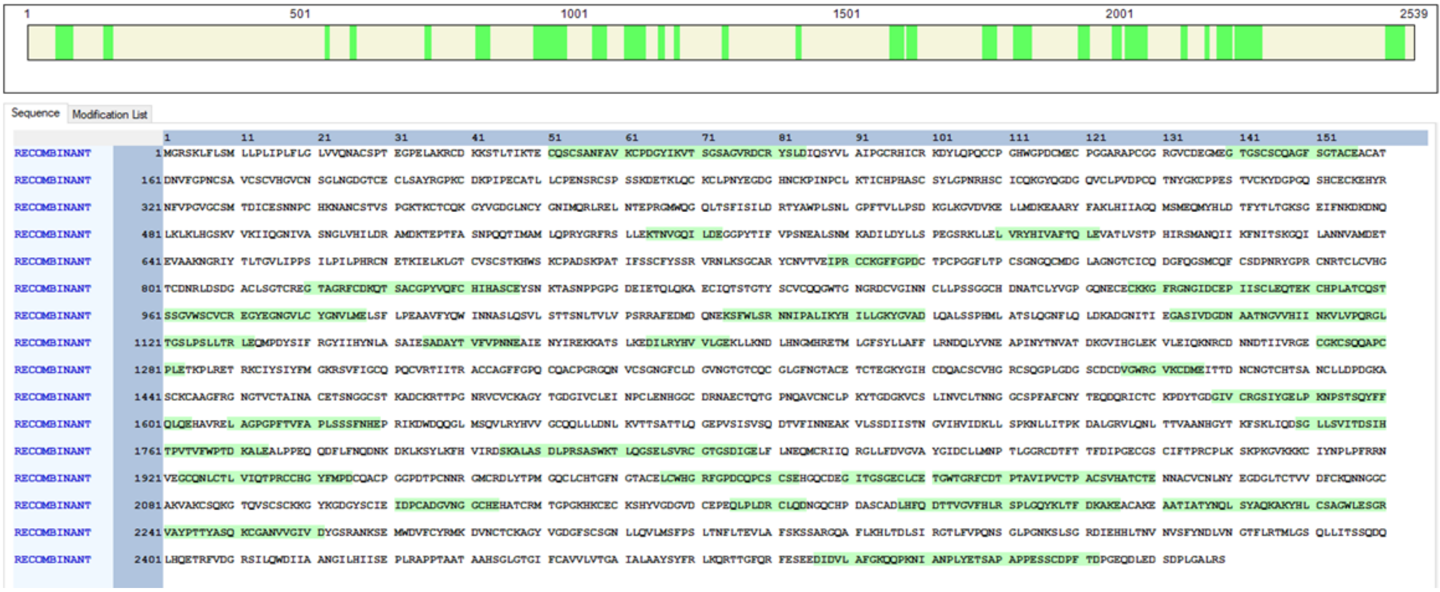

Fig. S3: Mass spectrometry coverage of the protein bands extracted from SDS-PAGE. V8 digest of the 260 and 230 kDa bands yielded a coverage of 31% and 24.5%, respectively. Sequences outlined in green represent the peptides that were identified by mass spectrometry. The bar just above the protein sequence is a representative coverage map of identified (green) protein.

Reduction/alkylation of 300HARE

M=marker, top band (170 kDa) just ran off bottom of gel
1=30 μg lysate
2=30 μg lysate, 1 μL DTT, 8 μL IA
3=30 μg lysate, 3 μL DTT, 8 μL IA
4=30 μg lysate, 6 μL DTT, 8 μL IA
5=30 μg lysate, 9 μL DTT, 8 μL IA
6=30 μg lysate, 12 μL DTT, 8 μL IA

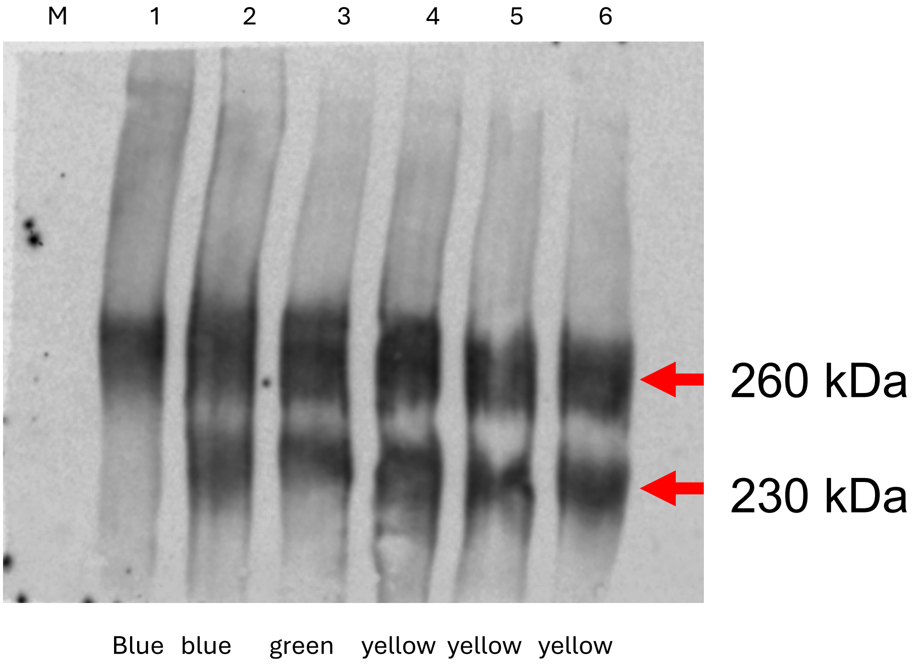

Fig. S4: Reduction of 300HARE: Thirty μg of cell lysate were treated with increasing concentration of DTT (1-12 mM) and 10 mM iodoacetamide (IA). Protein in lane 1 was not reduced. The color of the reaction mixture in Coomassie loading dye is indicated at the bottom of each lane of the gel.

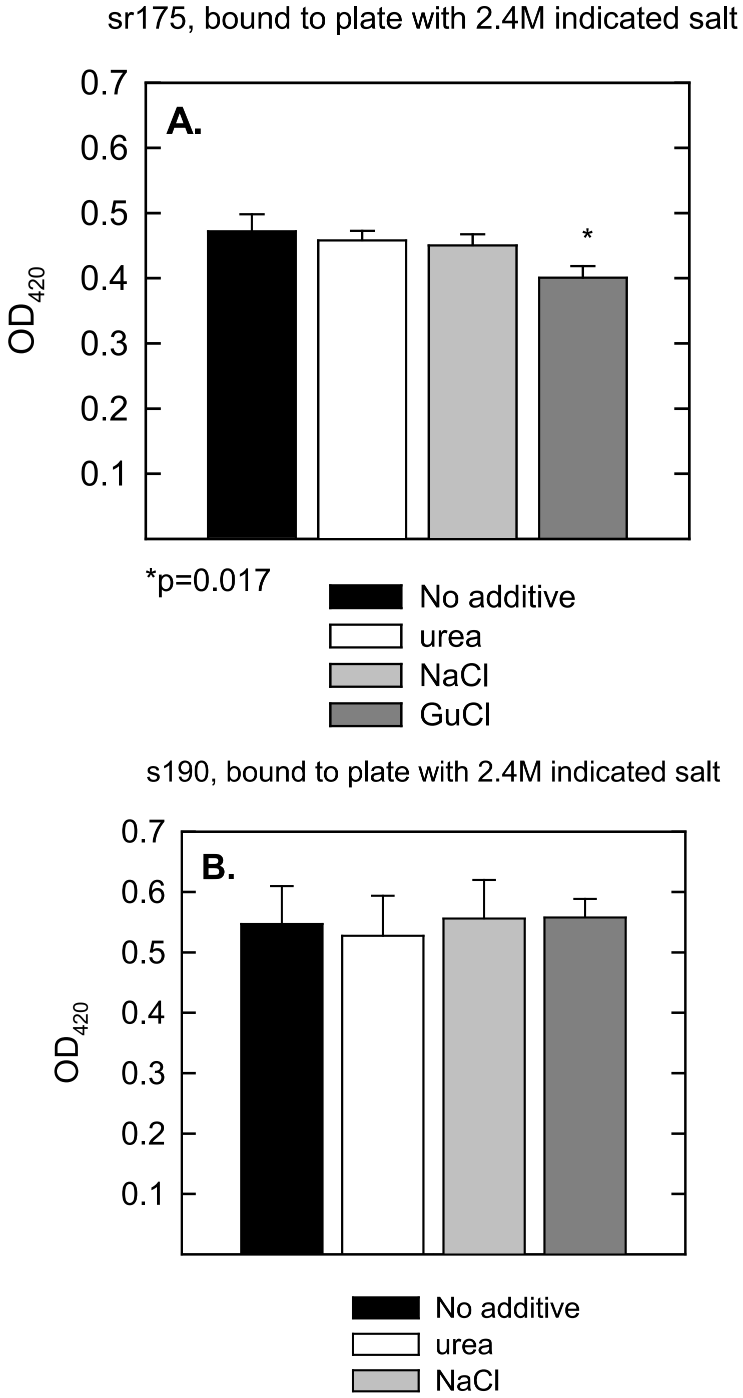

Fig. S5: Both (A) sr175 and (B) s190 were plated in F8 polysorp wells using plating buffer and blocked with blocking buffer and incubated in 2.4 M of the indicated salt. Protein was detected by anti-V5 primary antibody followed by anti-rabbit secondary antibody-AP. Color development was evaluated by a plate reader at A_{420}. n=4, *p=0.017.

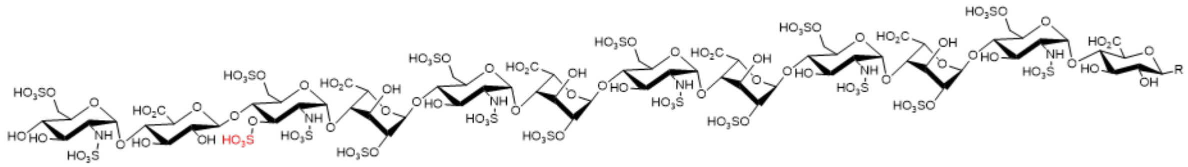

Fig. S6: Structure of Dekaparin. The lone 3-*O* sulfate group (3rd sugar from nonreducing end) is marked in red.

Fig. 1A

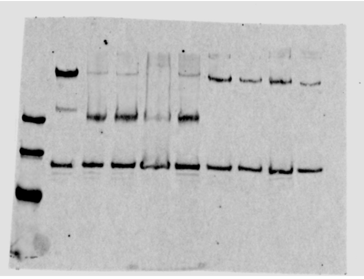

Fig. 1B

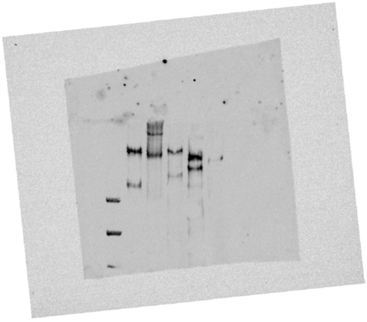

Fig. 1C

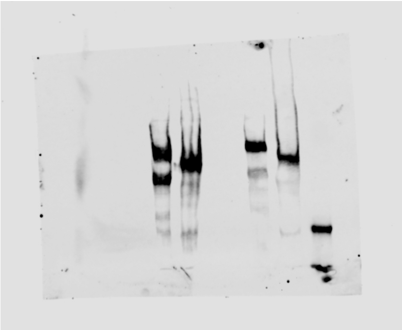

Fig. 2A

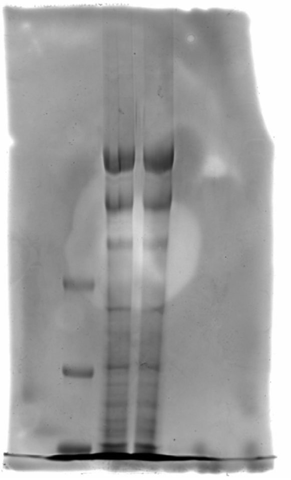

Fig. 3B

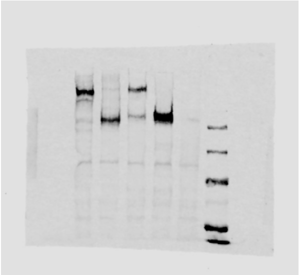

Fig. 6D

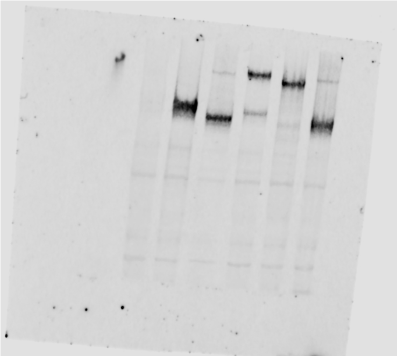

Fig. S4

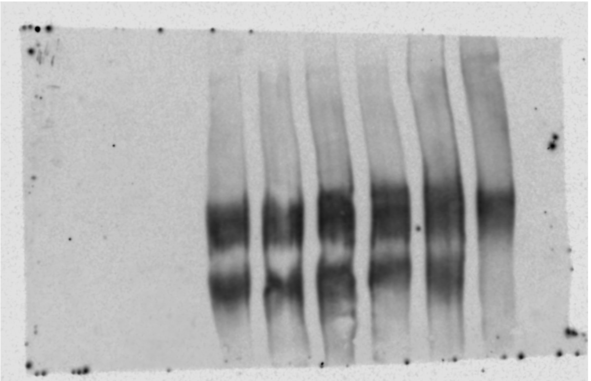

Fig. S7: Original images of all Western blots used in this manuscript. LiCor reagents were used for all blots in the infrared spectrum of 680 or 800 nm.
